# Human cadaver multipotent stromal/stem cells isolated from arteries stored in liquid nitrogen for 5 years

**DOI:** 10.1186/scrt397

**Published:** 2014-01-15

**Authors:** Sabrina Valente, Francesco Alviano, Carmen Ciavarella, Marina Buzzi, Francesca Ricci, Pier Luigi Tazzari, Pasqualepaolo Pagliaro, Gianandrea Pasquinelli

**Affiliations:** 1 DIMES – Department of Experimental, Diagnostic and Specialty Medicine, University of Bologna, Via Massarenti 9, 40138 Bologna, Italy; 2 DIMES – Department of Experimental, Diagnostic and Specialty Medicine, Unit of Histology, Embryology and Applied Biology, Via Belmeloro 8, 40138 Bologna, Italy; 3 Cardiovascular Tissue Bank – Immunohematology and Transfusion Medicine, University-Hospital St. Orsola-Malpighi, Polyclinic of Bologna, Via Massarenti 9, 40126 Bologna, Italy

## Abstract

**Introduction:**

Regenerative medicine challenges researchers to find noncontroversial, safe and abundant stem cell sources. In this context, harvesting from asystolic donors could represent an innovative and unlimited reservoir of different stem cells. In this study, cadaveric vascular tissues were established as an alternative source of human cadaver mesenchymal stromal/stem cells (hC-MSCs). We reported the successful cell isolation from postmortem arterial segments stored in a tissue-banking facility for at least 5 years.

**Methods:**

After thawing, hC-MSCs were isolated with a high efficiency (12 × 10^6^) and characterized with flow cytometry, immunofluorescence, molecular and ultrastructural approaches.

**Results:**

In early passages, hC-MSCs were clonogenic, highly proliferative and expressed mesenchymal (CD44, CD73, CD90, CD105, HLA-G), stemness (Stro-1, Oct-4, Notch-1), pericyte (CD146, PDGFR-β, NG2) and neuronal (Nestin) markers; hematopoietic and vascular markers were negative. These cells had colony and spheroid-forming abilities, multipotency for their potential to differentiate in multiple mesengenic lineages and immunosuppressive activity to counteract proliferation of phytohemagglutinin-stimulated blood mononuclear cells.

**Conclusions:**

The efficient procurement of stem cells from cadaveric sources, as postmortem vascular tissues, demonstrates that such cells can survive to prolonged ischemic insult, anoxia, freezing and dehydration injuries, thus paving the way for a scientific revolution where cadaver stromal/stem cells could effectively treat patients demanding cell therapies.

## Introduction

Regenerative medicine is a group of biomedical approaches based on cell therapies to solve the problem of the shortage of organ donors. For many diseases, stem cell therapy remains a possible alternative but requires a huge number of cells.

Human mesenchymal stromal/stem cells (hMSCs) are promising candidates for cell transplantation due to their ability for self-renewal, with a high growth rate, and their differentiation potential to produce mesodermal cell types such as adipocytes, osteocytes and chondrocytes [1]. Although human bone marrow is the best known source of hMSCs, the harvest is relatively invasive and stem cell numbers decrease significantly with donor age [2,3]. The search for an easily accessible source of hMSCs has led various research groups to explore numerous tissues, including arteries [4,5]. However, even if preferred for obvious ethical reasons, adult tissues can be limited in stem cell number when obtained from a living donor and thus the supply is also severely limited [6,7]. Research into an ideal hMSC source beside living donors is thus a true possibility that needs to be explored.

Cadaveric multiorgan donors are usually utilized to provide organ and tissue for transplants, but it is also reasonable to think that the same donors could represent an innovative and unlimited reservoir of different types of stem cells. Emerging evidence supported the idea that viable hMSCs can be isolated and expanded from cadaveric donors after postmortem intervals exceeding days [8-11]. Besides hematopoietic and neural stem cells, hMSCs derived from cadavers are also considered an encouraging source for potential cell-based therapies offering new hope of life after death [12]. The vascular wall has been described as a possible niche of vascular stem cells [4] but until now cadaveric vessels derived from nonheart-beating donors remain unstudied.

Postmortem human allografts are usually used in bypass surgery and peripheral vascular reconstruction in patients without sufficient autologous graft material [13]. In these cases, human vascular segments are cryopreserved for a long time in tissue-banking facilities to preserve functional characteristics and to guarantee a continuous availability of various-caliber segments for clinical application [14]. Due to the availability of adequate prosthetic material rapidly boosted in vascular surgery, many segments lie unused for years and unfortunately all of these potential stem cells sources are usually wasted.

In previous studies, we reported that fresh human vascular wall, harvested from heart-beating multiorgan donors, contains vascular stromal/stem cell progenitors that, *in vitro*, showed a high differentiation potential [4,5].

In this study, we demonstrate the successful isolation, propagation and morphological, phenotypic and functional characterization of human cadaver mesenchymal stromal/stem cells cells (hC-MSCs) derived by postmortem of variously sized arterial segments after 4 days from death of the donor and cryopreservation for more than 5 years.

## Materials and methods

### Cadaveric variously sized arteries

Donor tissues from Emilia Romagna Cardiovascular Tissue Bank facilities were used according to the policy approved by the local ethics committee (University-Hospital St. Orsola – Malpighi, Bologna, Italy; ethic project number APP-13-01) and informed consent was obtained from each donor before specimen collection. Human vascular tissues were explanted by the cardiovascular team from three young and healthy multi-tissue donors (nonheart-beating) 12 hours after death.

After collection, the arteries were kept in a sterile box with Celsior media (IMTIX SANGSTAT, Lyon, France), a flushing and cold storage solution for solid organ preservation, and were transferred to the Cardiovascular Tissue Bank at Bologna University-Hospital St. Orsola – Malpighi in isothermal boxes filled with ice within 4 hours after procurement. The artery segments were prepared, classified and transferred in an antibiotic mixture solution with RPMI 1640 (Cambrex Bio Science Vierviers, Vierviers, Belgium) for 72 hours at +4°C. Four days after donor death, the arteries were transferred into sterile bags containing 100 ml fresh cryoprotectant solution (RPMI 1640 with human albumin; Kedrion, Lucca, Italy) and Me_2_SO at a final concentration of 10%. The solution was cooled at 4°C for 30 minutes before its use. The bags were kept at 4°C for 30 minutes to allow the Me_2_SO to penetrate into the tissues completely. The bags were labeled and cryopreserved in liquid nitrogen vapor in a controlled rate freezing system (IceCube 1860; Sy-Lab, Wien, Austria) using an electronically monitored program that allows one to decrease the temperature at 1°C/minute to −45°C and at a faster rate thereafter until −120°C has been achieved. The cryopreserved arterial homografts were stored in the vapor phase of liquid nitrogen for 5 years. All donors were screened for virus markers. All procedures were performed in sterile conditions under a laminar flow cabinet [15].

### Isolation and cell culture

Segments of variously sized arteries with different embryological origin (epiaortic district and thoracic aorta) were obtained by postmortem human donors. The samples frozen for more than 5 years were dissociated by enzymic digestion with 0.3 mg/ml Liberase type II (Roche, Milan, Italy) in serum-free Dulbecco’s modified Eagle’s medium (DMEM; Lonza, Basel, Switzerland) overnight at 37°C using a rotor apparatus. After digestion, the homogenate was filtered through a cell strainer (Becton Dickinson, Franklin Lakes, NJ, USA), seeded at 1 × 10^5^/cm^2^ on collagen-I coated T75 flasks plates with smooth muscle growth medium-2 (Sm-GM2; Lonza) and incubated at 37°C in a humidified atmosphere with 5% CO_2_. Nonadherent cells were removed after 72 hours by washing with phosphate-buffered saline (PBS). Culture media was changed every 3 days until testing. When cells were near confluence, they were expanded *in vitro* for at least 14 passages. Before the isolation, a small piece of each vascular segment as well as the remaining digested tissue was fixed, hematoxylin and eosin stained and analyzed to verify the efficiency of the isolation method.

### Growth kinetics

All fresh isolated hC-MSCs were plated and then cultured until subconfluence. At each passage, viable cells were enumerated by trypan blue exclusion for evaluation of growth kinetics. The assessment of cell proliferation was performed for 3 weeks.

### Immunophenotyping

#### Flow cytometry

The hC-MSC immunophenotype was analyzed for the single expression of characteristic markers generally used to identify the hMSCs and stem cells using a flow cytometry analysis. To detect surface antigen, cells taken at passage 3 were washed twice with PBS and incubated for 20 minutes using the following extensive conjugated antibodies panel: anti-CD44-fluorescein isothiocyanate (FITC), anti-CD73-phycoerythrin (PE), anti-CD90-phycoerythrin-cyanine 5, anti-CD105-PE, anti-CD14-FITC, anti-CD31-PE, anti-CD34-FITC, anti-CD45-allophycocyanin, von Willebrand Factor (vWF; Dako Cytomation, Glostrup, Denmark), anti-CD146-PE, anti-platelet-derived growth factor (PDGF)-rβ (R&D Systems, Inc., Minneapolis, MN, USA), anti-NG2 (R&D Systems), anti-STRO-1 (R&D Systems), anti-Oct-4 (Santa Cruz Biotechnology, Santa Cruz, CA, USA), anti-Notch-1 (Santa Cruz Biotechnology) and HLA-G-FITC (Abcam, Cambridge, UK). The following secondary monoclonal antibodies (mAbs) were used after cell staining with unlabeled primary mAbs: anti-mouse IgG-allophycocyanin (Beckman-Coulter, Fullerton, CA, USA), anti-rabbit IgG-FITC (Dako, Glostrup, Denmark). To reveal vWF and Oct-4, the cells were fixed, permeabilized with the IntraPep Kit (Beckman-Coulter) and subsequently incubated with anti-mouse IgG-FITC (Dako). To study coexpression of CD73 and CD105 on CD34/CD45-negative hC-MSCs, cells were simultaneously incubated respectively with CD34-FITC, CD45-allophycocyanin, CD73-PE mAbs and CD34-FITC, CD45-allophycocyanin, CD105-PE mAbs. In addition, to verify the percentage of CD44^+^/CD90^+^ simultaneously expressing CD146 and PDGF-rβ, triple staining analyses were performed respectively with CD44-FITC, CD90-phycoerythrin-cyanine 5, PDGF-rβ conjugated with anti-mouse IgG-allophycocyanin and CD44-FITC, CD90-phycoerythrin-cyanine 5, CD146-PE mAbs. Negative controls were performed using appropriate conjugated irrelevant antibodies. Samples were analyzed using a Navios FC equipped with two lasers for data acquisition (Beckman-Coulter). Results were analyzed were elaborated with Kaluza FC Analysis software (Beckman-Coulter).

#### Immunofluorescence analysis

To detect intracytoplasmic antigens, an immunofluorescence staining was performed. Briefly, 4 × 10^4^ hC-MSCs were cultured on collagen biocoated slide chambers (BD Bioscence, San Jose, CA, USA) until near confluence. Subsequently, the samples were washed with PBS, followed by 2% paraformaldehyde plus 0.1% Triton X-100 for 4 minutes at room temperature. Fixed cells were then blocked in 1% bovine serum albumin in PBS solution for 30 minutes at room temperature and labeled for 1 hour at 37°C with primary antibodies. After repeated washing, the slides were incubated with Alexa Fluor 488 (1:250; Invitrogen, Carlsbad, CA, USA) secondary antibodies in 1% bovine serum albumin in PBS for 1 hour at 37°C in the dark. Finally, after several rinses, the samples were mounted and nuclei counterstained with Pro Long anti-fade reagent with DAPI (Molecular Probes, Milan, Italy). Primary antibodies and dilutions were used as follows: α-smooth muscle actin (1:9,000, Sigma, Saint Louis, Missouri, USA), calponin (1:40; Dako Cytomation), H-caldesmon (1:75; Dako), Desmin (1:300; Dako), Vimentin (1:100; Dako) and ki-67 (1:100; Novocastra, Wetzlar, Germany). In addition, the following neuronal markers were investigated: Neuron Specific Enolase (1:12,000; BioGenx, Fremont, CA, USA), Nestin (1:400; Millipore, Billerica, MA, USA), Neurofilament (1:100; Dako) and S100 (1:200; Dako). For a negative control, the samples were processed omitting the primary antibody, and no signal was detected. Images were taken on a Leica DMI4000 B inverted fluorescence microscope (Leica Microsystems, Milan, Italy) at × 20 magnification.

### Reverse transcriptase polymerase chain reaction gene expression analysis

Total RNA was extracted from hC-MSCs grown as an adherent monolayer and in suspension as spheres using RNA-extracting TRIreagent according to the manufacturer’s instructions (TRIzol reagent; Invitrogen). One microgram of total RNA was reverse transcribed in a 20 μl volume of reaction using a High Capacity Reverse Transcription Kit (Applied Biosystems, Carlsbad, CA, USA). All polymerase chain reaction (PCR) products were analyzed on 2% agarose gel electrophoresis with Tris-acetate–ethylenediamine tetraacetic acid buffer 1×, stained with ethidium bromide incorporation and photographed under ultraviolet light. β-Microglobulin was used as the housekeeping gene. A 100 base pair (bp) DNA ladder was loaded to allow PCR product size identification. The gel was subjected to electrophoresis at a constant 100 V for 45 minutes. Genes and respective primers are presented in Table 1. The PCR primers were purchased from Invitrogen. β2-Microglobulin was used as the housekeeping gene to value the cDNA quality.

**Table 1:** Reverse transcriptase polymerase chain reaction: primers and conditions

**Gene**	**Primer sequence**	**Amplicon length (base pairs)**	**T (°C)**
β-Microglobulin	Reverse: 5′-ATCTTCAAACCTCCATGATG-3′	114	58
	Forward: 5′-ACCCCCACTGAAAAAGATGA-3′		
SOX2	Reverse: 5′-GCGCCGCGGCCGGTATTTAT-3′	208	61
	Forward: 5′-CCGGCGGCAACCAGAAGAACAG-3′		
c-KIT	Reverse: 5′-CATACAAGGAGCGGTCAACA-3′	275	67
	Forward: 5′-GTCTCCACCATCCATCCATC-3′		
OCT-4	Reverse: 5′-CCACATCGGCCTGTGTATAT-3′	380	62
	Forward a: 5′-CTCCTGGAGGGCCAGGAATC-3′	402	
	Forward b: 5′-ATGCATGAGTCAGTGAACAG-3′		
NOTCH-1	Reverse: 5′-TGGCATCAGCTGGCACTCGTCC-3′	496	67
	Forward: 5′-CCGGCTGGTCAGGGAAATCGTG-3′		
KDR	Reverse: 5′-TTTGTCACTGAGACAGCTTGG-3′	555	61
	Forward: 5′-TATAGATGGTGTAACCCGGA-3′		
PPAR-γ	Reverse: 5′-ACAGTGTATGAGTGAAGGAAT-3′	101	54.5
	Forward: 5′-CAGTGTGAATTACAGCAAACC-3′		
Osteocalcin	Reverse: 5′-TCAGCCAACTCGTCACAGTC-3′	175	58
	Forward: 5′-GTGCAGAGTCCAGCAAAGGT-3′		
Osteopontin	Reverse: 5′-GTCATGGCTTTCGTTGGACT-3′	200	58
	Forward: 5′-TTGCAGTGATTTGCTTTTGC-3′		
RUNX2	Reverse: 5′-GACTGGCGGGGTGTAAGTAA-3′	161	58
	Forward: 5′-TCTGGCCTTCCACTCTCAGT-3′		
Type II collagen	Reverse: 5′-GGGGGTCCAGGGTTGCCATTG-3′	352	58
	Forward: 5′-ACGGCGAGAAGGGAGAAGTTG-3′		

### *In vitro* spheroid formation

To determinate whether hC-MSCs have the ability to grow forming spheres in nonadherent conditions, cells taken at passage 3 were filtered through a cell strainer to obtain a single cell suspension and plated at density of 3 × 10^4^ cells/well in ultralow attachment 24-well plates. After few days, cell aggregation in spheroids was observed under light microscopy (LM) and processed for gene expression analysis as described previously.

### Clonogenic assay

To assess the self-renewal capacity, passage 3 hC-MSCs were trypsinized, counted and plated in 96-well plates at a limiting dilution of 0.3 cells/100 μl concentration to have a single clone per well. During the culture, each well was daily examined for colony formation and photographed under LM at × 4 magnification. Each test was performed in triplicate. After 1 month, confluent wells were counted to determine the number of produced colonies.

### Multilineage differentiation potential

hC-MSCs taken at passage 3 were differentiated towards mesodermal lineages: adipogenesis, osteogenesis, chondrogenesis, leiomyogenesis and angiogenesis.

#### Adipogenic potential

Adipogenesis was induced by plating hC-MSCs at density of 6 × 10^4^ cells/well in a 24-well plate using the Mesenchymal Stem Cell Adipogenesis Kit (Chemicon International, Temecula, CA, USA) according to the manufacturer’s instructions. Induction medium was replaced every 2 to 3 days for 2 to 3 weeks and alternated with maintenance medium (DMEM 10% fetal bovine serum (FBS) and 0.02 mg/ml insulin). Three complete cycles of induction/maintenance medium stimulated optimal adipogenic differentiation, forming adipocytes. Control cells were culture in basal medium (DMEM plus 10% FBS). To confirm their identity, hC-MSCs were fixed and the cytoplasmic presence of lipid droplets was assessed by Oil Red O staining and transmission electron microscopy (TEM). The cells cultured were also processed for reverse transcriptase (RT)-PCR analysis as specified above to investigate the expression of adipogenic transcription factor peroxisome proliferator-activated receptor gamma (PPARγ).

#### Osteogenic potential

Osteogenesis was induced by plating hC-MSCs at density 6 × 10^4^ cells/well in a 24-well plate using the osteogenic induction medium Mesenchymal Stem Cell Osteogenesis Kit (Chemicon International) with the addition of 10% FBS, maintained for 3 weeks, replacing the medium every 2 to 3 days, according to the manufacturer’s instructions. Control cells were cultured in basal medium (DMEM plus 10% FBS). All experiments were followed by morphological evaluation by LM. To detect mineral deposition, the cells were fixed and assessed by Alizarin Red staining and TEM investigation. The cells cultured were also processed for RT-PCR analysis as specified above to investigate the expression of osteogenic markers Osteocalcin, Osteopontin and RUNX-2.

#### Chondrogenic potential

Aliquots of 2.5 × 10^5^ cells were pelleted in polypropylene conical tubes in differentiation basal medium chondrogenic (Poietics, Lonza) supplemented with hMSC Chondrogenic Single Quotes (Poietics, Lonza) and 10 ng/ml transforming growth factor beta-3 (SIGMA, Lonza). This medium was replaced every 2 to 3 days for 3 weeks. Control cells were cultured in the same differentiation medium without transforming growth factor beta-3. Pellets were formalin fixed, paraffin embedded and stained with Alcian Blue and PAS using a standard method. Immunostaining for type II collagen (1:200; Chemicon Millipore, Billerica, MA, USA) using a nonbiotin-amplified method (NovoLink Polymer Detection Kit; Novocastra, Newcastle upon Tyne, UK) was performed according to manufacturer’s instructions. Images were acquired using Image-Pro PlusW 6 software (v. 4.5 [16]; MediaCybernetics, Rockville, MD, USA) at × 20 magnification. All samples were also analyzed by TEM to evaluate proteoglycan synthesis. To investigate the expression of chondrogenic marker type II collagen, 10 consecutive 10-μm-thick sections from the same samples used for the chondrogenic assays were processed for RT-PCR using the RNeasy® Formalin-Fixed, Paraffin-Embedded kit (Qiagen, Valencia, CA, USA) according to the manufacturer’s instructions.

#### Smooth muscle cell differentiation

Cells (15 × 10^3^ cells/well) were seeded in a six-well plate in SmGM-2. After 24 hours, the medium was changed for induction medium containing SmGM-2 plus 10 ng/ml transforming growth factor beta-1 and 5 ng/ml PDGF-BB (all growth factors from Sigma). The medium was changed every 3 days and the induction period lasted for 7 days. Control cells were cultured in SmGM-2 without additional growth factors. At the end of differentiation, hC-MSCs were fixed and resin embedded for TEM analysis to disclose contractile filaments induction and organization.

#### Angiogenic potential

The ability to form capillary-like tubes was tested in a semisolid matrix. Briefly, hC-MSCs taken at passage 3 were cultured at confluence for 7 days in DMEM plus 2% FBS with 50 ng/ml vascular endothelial growth factor (VEGF; Sigma). Control cells were culture in basal medium (DMEM plus 10% FBS). At the end of induction, 5 × 10^3^ hC-MSCs were plated onto the Matrigel (BD Bioscence) solution, solidified and incubated at 37°C 5% CO_2_. Human umbilical vein endothelial cells were used as a positive control. The formation of capillary-like structures was observed using LM after 2, 4 and 6 hours. In parallel experiments, the induced and control hC-MSCs were analyzed at flow cytometry for the expression of vWF and CD31 endothelial markers.

### Transmission electron microscopy

For TEM, pellets of uninduced and induced hC-MSCs were washed with phosphate buffer, fixed for 24 hours at 4°C in Karnowsky fixative (2% glutaraldehyde, 4% formaldehyde in 0.1 M phosphate buffer), post-fixed in 1% buffered osmium tetroxide for 1 hour at room temperature, dehydrated through graded ethanol, followed by propylene oxide, and embedded in Araldite resin. Ultrathin sections were transferred to specimen support grids and were counterstained with uranyl acetate and lead citrate prior to examination in a Philips 400 T transmission electron microscope (FEI Company, Milan, Italy).

### Immunomodulatory assay

To test the immunomodulatory activity, hC-MSCs at passage 3 were trypsinized and plated at a density of 25 × 10^3^ cells/cm^2^ in a six-well plate (*n* = 3). They were then cocultured with peripheral blood mononuclear cells (PBMCs), derived from healthy volunteer donors of the Transfusion Medicine Service, Bologna University-Hospital St. Orsola – Malpighi (according to the policy of the local ethical committee). PBMCs were isolated by density gradient centrifugation and plated on the hC-MSC monolayer at a density of 2.5 × 10^6^ cells/well in RPMI 1640 (Lonza, Walkersville, MD, USA). PBMCs were activated by addition of phytohemagglutin (PHA, 5 g/ml; Sigma-Aldrich, Saint Louis, Missouri, USA) and incubated for 72 hours at 37°C, 5% CO_2._ PBMCs were fixed with 70% ethanol at 4°C, stained with propidium iodide (Beckman Coulter) at room temperature for 10 minutes and analyzed by flow cytometry.

### Statistical analysis

The results are presented as the mean (from the indicated number of samples) ± standard deviation. Two-tailed *t* tests were conducted to determine statistical significance.

## Results

### Human cadaver mesenchymal stromal/stem cell isolation, early characterization and expansion

**Figure 1:**
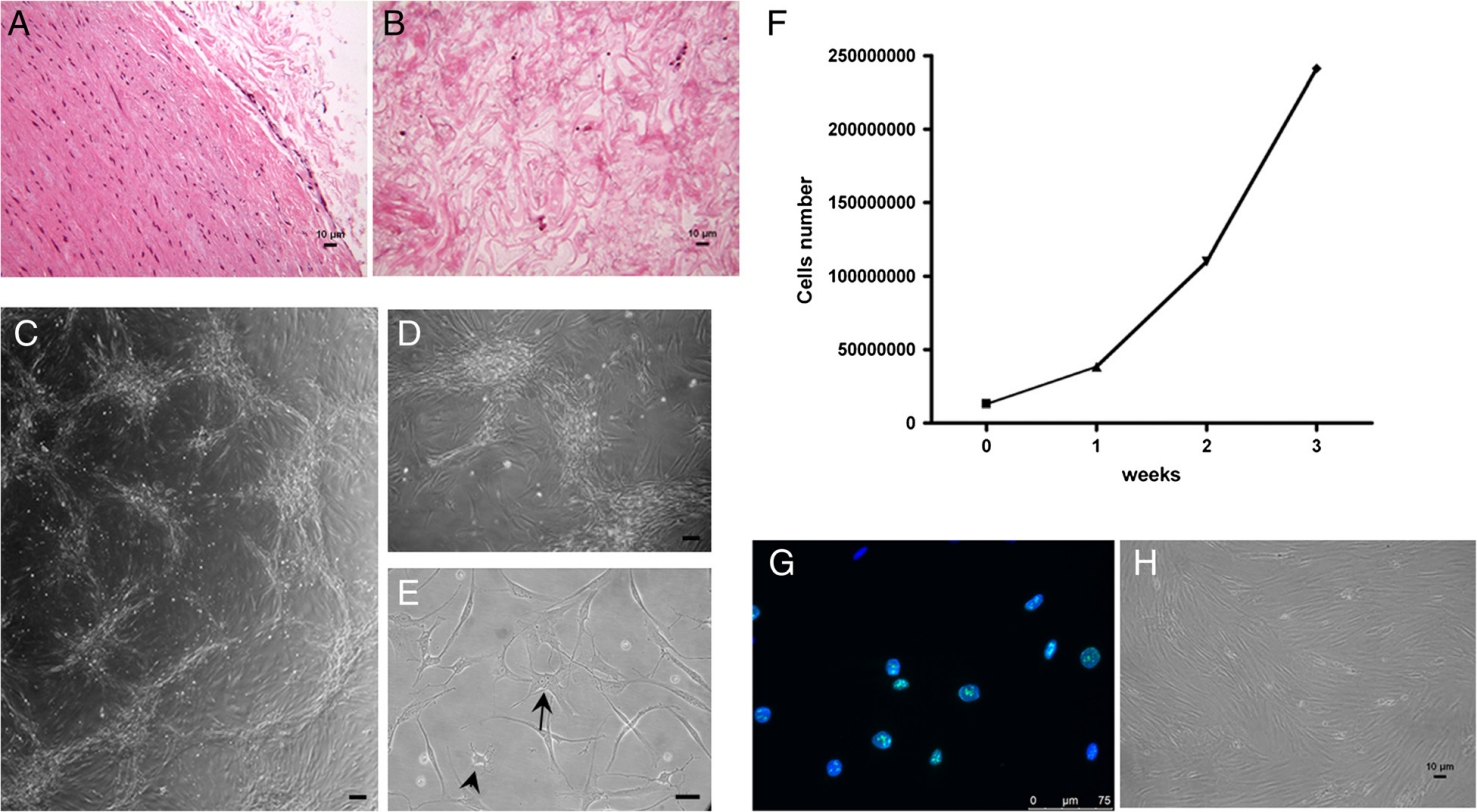
**Human cadaver mesenchymal stromal/stem cell isolation, early characterization and expansion.** Representative histological staining of native **(A)** and digested arterial tissue **(B)** after enzymatic isolation of human cadaver mesenchymal stromal/stem cells (hC-MSCs) (scale bars =10 μm). **(C)**, **(D)** After harvesting, hC-MSCs collected from three postmortem artery segments show clonogenic activity (scale bars = 50 μm). **(E)** Numerous poly-nucleated cells (arrow), spindle-shaped cells, dendritic (arrowhead) cells and rounded cells (scale bar = 20 μm). **(F)** hC-MSC growth kinetics. After 3 weeks of culture, the cells seeded were expanded approximately 20-fold and yielded 250 × 10^6^ cells. **(G)** ki-67 nuclear immunoreactivity (scale bar = 75 μm). **(H)** The hC-MSCs at passage 3 became elongated and spindle-shaped with long and thin cytoplasmic projections (scale bar =10 μm).

hC-MSCs were successfully isolated and expanded *in vitro* from three human cadaver arterial allografts after 4 days postmortem and more than 5 years of liquid nitrogen bank storage. After cell recovery, histological observation of the residual arterial tissue revealed that the tissue architecture and tunica layering were no longer distinguishable while only rare cells still remained enclosed in the native tissue (Figure 1A, B). The initial cell number recovered was overall 4 × 10^5^ cells/cm^2^. These results documented the good efficiency of the isolation procedure. In early passages (<3), these cells, showing strong plastic adhesion, formed small colonies that rapidly became confluent, giving origin to a vorticous and intersecting pattern suggesting an innate clonogenic ability (Figure 1C, D); numerous poly-nucleated cells (one out of 20 cells each 100× microscopic field) with two, three or more nuclei were also evident; most of the adherent cells had a spindle-shaped appearance; dendritic and rounded cells were also seen (Figure 1E). hC-MSCs were long-lived in culture, highly proliferating and exhibited evidence of ongoing cell division. We tested the cells for up to 14 passages without losing their proliferative capacity. The cell proliferation rate of hC-MSCs was determined by evaluating the total number of hC-MSCs at initial seeding and after 3 weeks of subconfluent culture condition; the total cell count was performed with a hemocytometer and trypan blue exclusion. As shown in Figure 1F, 12 × 10^6^ freshly derived hC-MSCs were expanded approximately 20-fold in 3 weeks and yielded 250 × 10^6^ cells. The ki-67 nuclear immunoreactivity demonstrated that more than 90% of the overall seeded cells were cycling (Figure 1G). After the passage 3, the starry-like appearance of cell culture became lost and more classic growth pattern was seen; hC-MSCs were elongated and homogeneously spindle-shaped in morphology with thin cytoplasmic projections (Figure 1H).

### Human cadaver mesenchymal stromal/stem cell phenotypic and molecular characterization

**Figure 2:**
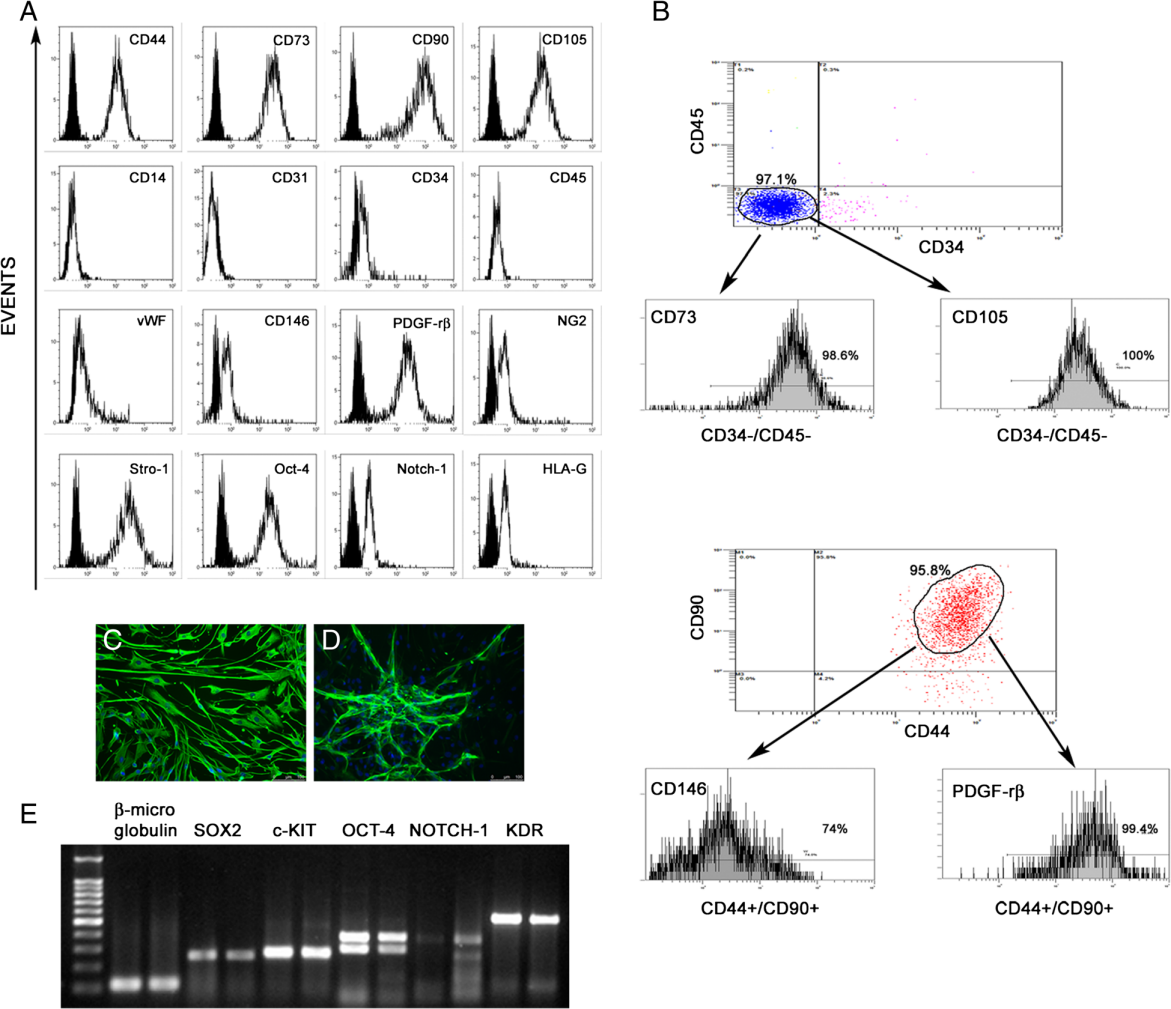
**Human cadaver mesenchymal stromal/stem cell phenotypic and molecular characterization. (A)** Representative flow cytometry analysis of mesenchymal, pericytic, stem cell, hematopoietic and vascular markers. Isotype controls are presented as filled black histograms, the specific cell markers as white histograms. **(B)** Flow cytometry analysis of hematopoietic, mesenchymal and pericyte marker coexpressions. Percentage and cytograms from a representative experiment. Immunofluorescence staining for Vimentin **(C)** and Nestin **(D)** in human cadaver mesenchymal stromal/stem cells (hC-MSCs). Nuclei were counterstained with DAPI (blue) and cell positive in green. Scale bars = 100 μm. **(E)** Stem cell gene expression analysis of SOX2, c-KIT, OCT-4, NOTCH-1 and KDR. β-Microglobulin was used as the housekeeping gene. The far left lane contains a 100 base pair ladder.

At the third replaying, flow cytometry analysis showed that hC-MSCs expressed recognized markers of hMSCs (CD44, CD73, CD90 and CD105), pericyte antigens (CD146, PDGF-rβ and NG2) and stemness markers (Stro-1, Oct-4 and Notch-1). On the contrary, no cells expressed markers of hematopoietic lineage (CD14 and CD45), hematopoietic progenitor (CD34) or endothelial cells (CD31, vWF). The isolated cells also constituting expressed of HLA-G antigen, a well-known tolerogenic molecule involved in the immuomodulatory activity of mesenchymal stromal/stem cells [17] (Figure 2A). Triple flow cytometry immunostaining of hC-MSCs revealed that 98.6% of CD34^–^/CD45^–^ were CD73^+^ and 100% of CD34^–^/CD45^–^ were CD105^+^. Regarding pericyte antigens, 99.4% of CD44^+^/CD90^+^ coexpressed PDGF-rβ and 74% of CD44^+^/CD90^+^ stained with CD146 (Figure 2B). In addition to flow cytometry analysis, a single immunofluorescence staining was performed to investigate the smooth muscle (α-smooth muscle actin, calponin, h-caldesmon, Vimentin and Desmin) and neural (NSE, Nestin, Neurofilament and S100) phenotypes. The intermediate filaments Vimentin and Nestin were strongly expressed virtually in all cells (Figure 2C, D), whereas Neurofilament was positive in rare cells. The remaining markers were negative. Gene expression analysis performed at passage 3 revealed that hC-MSCs constitutively expressed high transcripts associated with similar stemness status as SOX2, c-KIT, the two isoforms of OCT-4 (380 bp, 308 bp) and KDR, while NOTCH-1 mRNA levels were lower (Figure 2E). All genes investigated are expressed in embryonic stem cells and are involved in survival and proliferation/differentiation pathways.

### Human cadaver mesenchymal stromal/stem cell stemness properties

**Figure 3:**
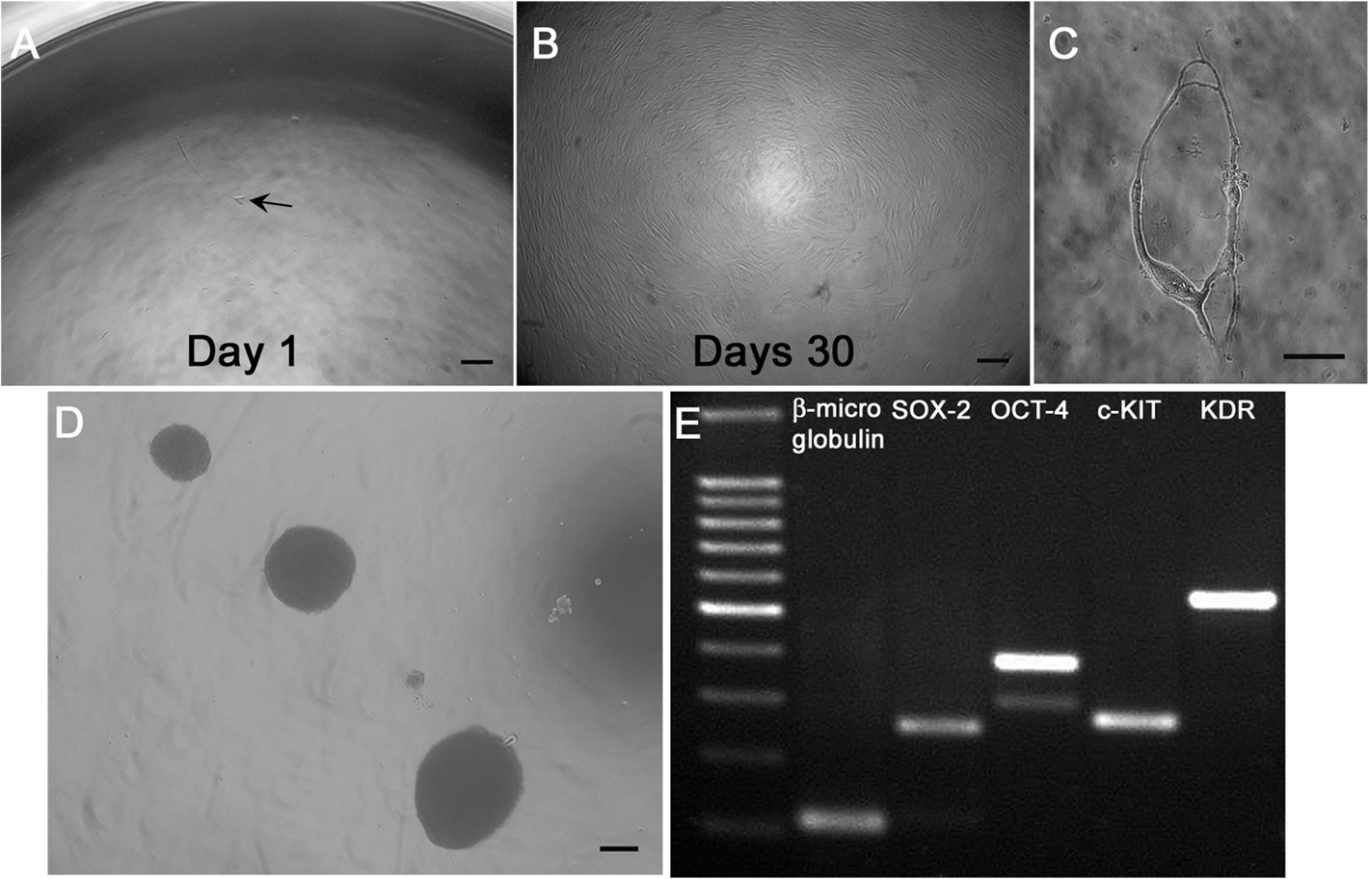
**Human cadaver mesenchymal stromal/stem cell stemness property.** Clone-forming potential of **(A)** a single seeded human cadaver mesenchymal stromal/stem cell (hC-MSC) (arrow) that **(B)** reached the confluence after 30 days of culture (scale bars = 50 μm). **(C)** Nonclonogenic single cell with a ring-shaped morphology (scale bar = 10 μm). **(D)** When plated in nonadhesion conditions, free floating spheres are generated (scale bar = 50 μm). **(E)** Reverse transcriptase polymerase chain reaction analysis performed on spheroids showed expression of SOX2, OCT-4, c-KIT and KDR. β-Microglobulin was used as the housekeeping gene. The far left lane contains a 100 base pair ladder.

hC-MSCs showed a prominent clonogenic capacity; after seeding, one seeded cell was observed in numerous wells and was followed during all culture periods (Figure 3A). Ten days later, a sporadic group of eight to 10 cells appeared in about 3% of the seeded wells; at day 30, a relevant increase of wells (about 8% of the total seeded wells) displayed clonogenic growth (Figure 3B). Interestingly, sporadic single cell clones showed no clonogenic potential and exhibited a ring-shaped morphology generated by the extrusion of long and thin cell processes that bent, forming circular profiles (Figure 3C).

hC-MSCs also showed the ability to form spheroids when grown in nonadherent conditions; after 5 days of culture they generated multiple spheroids that resembled embryo-like bodies when observed in plate using an inverted LM (Figure 3D). Molecular analysis by RT-PCR showed expression of SOX2, OCT-4, c-KIT and KDR (Figure 3E).

### Human cadaver mesenchymal stromal/stem cell mesengenic potential

hC-MSCs were cultured in appropriate culture conditions to test their tripotential commitments including adipogenic and osteo-chondrogenic lineages. Leiomyogenic and angiogenic potentials were also explored.

**Figure 4:**
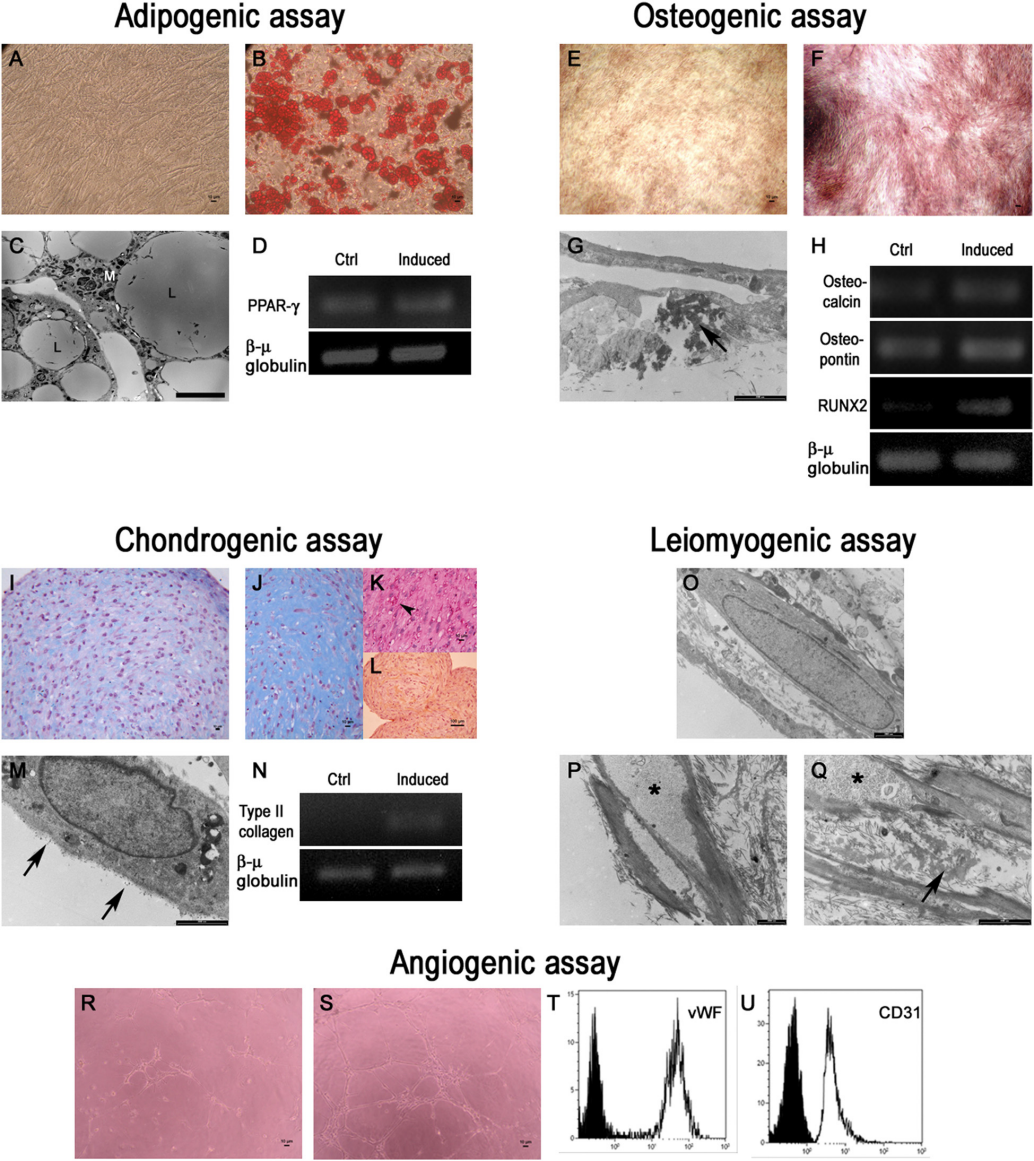
**Human cadaver mesenchymal stromal/stem cell mesengenic potential. (A)** Control human cadaver mesenchymal stromal/stem cells (hC-MSCs) did not display cytoplasm lipid drops. **(B)** Oil Red O stained adipocytic multivacuolar cells in red. (A), (B) Scale bars = 10 μm. **(C)** Transmission electron microscopy (TEM) showed multiple lipid vacuoles and small dense mitochondria in the cytoplasm. L, lipid droplets; M, mitochondria. Scale bar = 2 μm. **(D)** Reverse transcriptase polymerase chain reaction of peroxisome proliferator-activated receptor gamma (PPARγ) expression. β-Microglobulin was used as the housekeeping gene. **(E)** Control hC-MSCs did not display calcium deposition in the extracellular matrix. **(F)** Alizarin Red stained calcium deposits. (E), (F) Scale bars = 10 μm. **(G)** TEM confirmed the presence of osteoid matrix and needle-shaped hydroxyapatite crystals (arrow). Scale bar = 2 μm. **(H)** Gene expression analysis of Osteocalcin, Osteopontin and RUNX-2. β-Microglobulin was used as the housekeeping gene. **(I)** Control hC-MSCs did not display proteoglycan-rich extracellular matrix. **(J)** Alcian Blue stained proteoglycan-rich extracellular matrix. **(K)** Glycogen inclusions (arrow) stained by PAS staining with and without diastase pretreatment. (I), (J), (K) Scale bars = 10 μm. **(L)** Human collagen type II immunostaining positive in the extracellular matrix. Scale bar = 100 μm. **(M)** TEM analysis revealed proteoglycans adherent to the cell membrane (arrows). Scale bar = 2 μm. **(N)** Molecular analysis of type II collagen transcript expression. β-Microglobulin was used as the housekeeping gene. **(O)** Control hC-MSCs did not display contractile filaments. **(P)** TEM analysis revealed peripherally arranged contractile filaments, dense bodies, glycogen deposits (*) and profiles of rough endoplasmic reticulum. **(Q)** Elastic lamellae in the extracellular matrix (arrow). O), (P), (Q) Scale bars = 2 μm. Matrigel assay in the absence **(R)** and presence **(S)** of vascular endothelial growth factor (VEGF; 50 ng/ml for 7 days) after 6 hours. (R), (S) Scale bars = 10 μm. **(T)**, **(U)** Flow cytometry analysis for von Willebrand factor (vWF) and CD31 expression in hC-MSCs cultured in the absence and in the presence of VEGF. Uninduced cells are presented as filled black histograms, differentiated cells as white histograms.

Adipogenic differentiation was successful and confirmed by Oil Red O staining and ultrastructural analysis. hC-MSCs showed multiple lipid-rich vacuoles in the cytoplasm that increased in size and number with the time of induction and were intensely stained red (Figure 4B). TEM revealed confluent lipid droplets, small dense mitochondria and intense endocytic activity (Figure 4C). RT-PCR showed the upregulation of PPARγ, a critical player of adipocyte differentiation (Figure 4D). Osteogenic differentiation was confirmed by Alizarin Red staining and ultrastructure. The differentiation was noted as early around 10 days of induction by morphological changes and, at the end of the induction period, by calcium accumulation (Figure 4F). TEM revealed in the extracellular space moderately to electron dense fibrillary deposits that were decorated with needle-shaped hydroxyapatite crystals (Figure 4G). RT-PCR showed that Osteocalcin, Osteopontin and RUNX-2 increased transcript expression (Figure 4H). All genes investigated are expressed in osteogenic differentiation pathways.

Chondrogenic differentiation was documented using Alcian Blue dye, human collagen type II immunostaining and ultrastructure. During the induction, matrix changes in micromass cell culture were noted and, at the end of the induction period, alcianophilia in proteoglycan-rich extracellular matrix was seen (Figure 4J). Changes in the extracellular matrix were accompanied by the presence of clear vacuoles in the cell cytoplasm that PAS staining with and without diastase pretreatment showed to be glycogen inclusions (Figure 4K). Immunohistochemistry analysis revealed, in the extracellular matrix, the diffuse presence of human type II collagen (Figure 4L), a specific marker for chondroblasts, which is typically found in joint cartilage. Ultrastructural analysis performed at the periphery of the cell micromass showed proteoglycan particles adherent to the cell membrane (Figure 4M). RT-PCR showed type II collagen mRNA expression (Figure 4N). Leiomyogenic differentiation was analyzed by TEM. At the end of induction, ultrastructural features were peripherally arranged contractile filaments with subplasmalemmal linear densities and dense bodies, glycogen deposits and profiles of rough endoplasmic reticulum; in the extracellular matrix, elastic lamellae were seen (Figure 4P, Q). All mesodermal commitment controls retained their morphology and did not display cytoplasm lipid vacuoles (Figure 4A), calcium deposition in the extracellular matrix (Figure 4E), proteoglycan-rich extracellular matrix (Figure 4I) and contractile filaments (Figure 4O).

Angiogenic differentiation was evaluated using a semisolid matrix assay. After 6 hours, the uninduced hC-MSCs organized themselves into a few capillary structures and most of the cells remained scattered in the medium (Figure 4R). When cultivated in the presence of VEGF, the cells rapidly aligned themselves, formed hollow tube-like structures with thin cytoplasmic projections sprouting from the cell periphery and appeared connected by thicker projections forming an evident capillary-like network (Figure 4S). Flow cytometry analysis showed that vWF and CD31, markers of mature endothelium, were clearly promoted by VEGF (Figure 4T, U). On the contrary, human umbilical vein endothelial cells, used as positive control, spontaneously aggregated in a capillary-like network when seeded on Matrigel (data not shown).

### Human cadaver mesenchymal stromal/stem cell immunomodulatory ability

**Figure 5:**
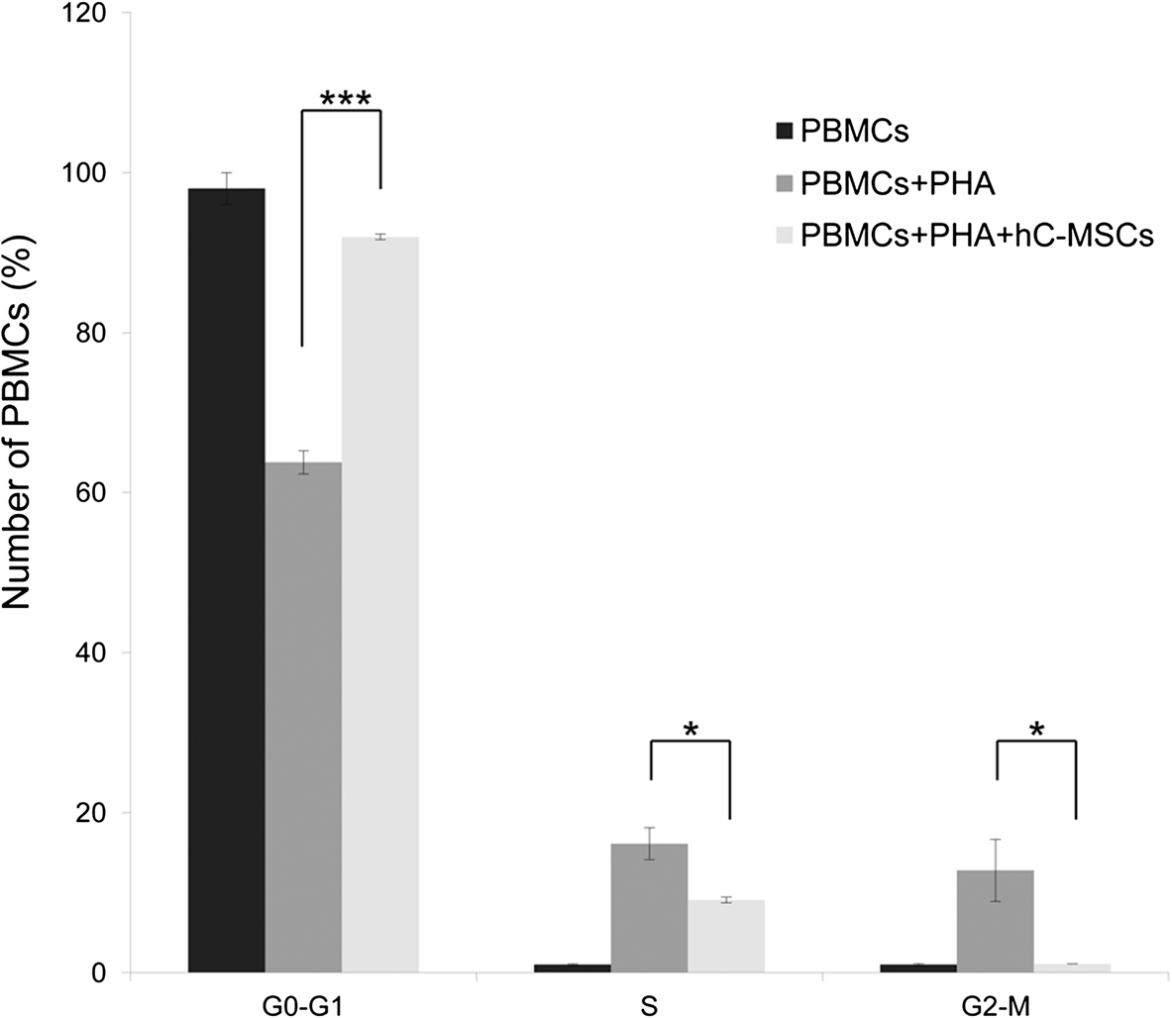
**Human cadaver mesenchymal stromal/stem cell immunomodulatory ability.** Human cadaver mesenchymal stromal/stem cell (hC-MSC) immunosuppressive effect on activated peripheral blood mononuclear cells (PBMCs). Analysis of the PBMC distribution in cell cycle phases after coculture with hC-MSCs. Phytohemagglutin (PHA)-activated PBMCs reduced proliferation when co- = cultured with hC-MSCs. Data expressed as a percentage of PBMCs for each cell cycle phase. Mean ± standard deviation; *n* = 3. *P <0.05; ***P <0.001.

To test whether hC-MSCs exert an immunomodulatory effect on co-cultured PHA-stimulated PBMC proliferation, the PBMC distribution in the cell cycle phases was evaluated (Figure 5). In three independent experiments we observed that unstimulated PBMCs were all in the G_0_/G_1_ phase, while activated PBMCs without hC-MSC co-culture were 63.8 ± 2.1% in the G_0_/G_1_ phase_,_ 16.1 ± 2.9% in the S phase and 12.8 ± 3.9% in the G_2_ phase. When hC-MSCs were present in coculture, we observed a significant increase of PBMCs in the G_0_/G_1_ phase (92 ± 0.5%) and an equally significant reduction of PBMCs in the S and G_2_/M phases, respectively 9.1 ± 0.4% and 0.2 ± 0.1%. These results suggested that hC-MSCs have an immunomodulatory effect on stimulated PBMCs mediated by cell cycle arrest.

## Discussion

The self-renewal and multilineage potential of hMSCs have generated a growing interest about potential application of these cells in tissue regeneration and cell-based therapies. In addition to bone marrow, various human tissues have also been reported to contain hMSCs capable of multilineage differentiation [5,7,18-21]. However, hMSCs are extremely rare and available in very low numbers when recovered by biopsy material [6,7], and *in vitro* expansion is required to achieve the necessary cell number useful for clinical applications [2]. To match the demand for regenerative medicine it is necessary to find an available and unlimited reservoir of hMSCs. Bone marrow transplantation [9] as well as pancreatic islets [22] from cadaveric donors have been reported in several studies as a clinical reality. Asystolic, cadaver donors are utilized to provide multiple organs and tissues for transplants, and stem cell retrieval from these sources could represent an original and noteworthy choice. Cadaver donors may become intriguing and advantageous, promising stem cell reservoirs due to restricted ethical issues, availability of tissues and cells; moreover, written consent procurement and safety collection procedures are extensively implemented. Furthermore, differentiated cells in the cadaver die in a couple of days, whereas stem cells such as mesenchymal stromal/stem cells residing in a hypoxic niche can survive and be harvested even after death. These quiescent or dormancy stem cells survive by adapting to low oxygen consumption with a slow metabolism and no transcription program [23]. The isolation of viable and functional cadaveric stem cells from human bone marrow [24,25], brain [10,26] and muscle [12] up to many days postmortem is possible today, but cadaveric cryopreserved vessels remain unstudied.

Cadaveric human vessels are usually used as a bypass in peripheral vascular disease [27]. After clinical suitability, human vascular segments are stored at deep subzero temperatures, allowing preservation of vascular tissues for a virtually infinite time [28] in banking facilities [29]. With the use of adequate prosthetic materials rapidly boosted in vascular surgery, many cryopreserved banked arterial segments remain unused and are destined to discharge after 5 years of storage; any viable and functional cell is therefore usually wasted. These facts justify our research on the use of long-time frozen arteries as an alternative and unlimited source of these cells for allogenic use.

In this study, cell isolation, expansion, morphological, phenotypic and functional characterization of hC-MSCs was carried out successfully from human vascular segments after 4 days from the death of donor and cryopreserved for more than 5 years. We showed that hC-MSCs can persist after prolonged ischemic insult and can survive for extended postmortem periods and long-time cryopreservation without losing their stemness features. We believe that anoxia, the lack of nutrients, cryogenic stress and tissue dehydration/rehydration, and other postmortem factors might contribute to selecting only the more robust and undifferentiated stem cells over the more differentiated cells from tissues in living donors.

We successful isolated a cell population that displayed morphological characteristics, immunophenotypic markers and differentiation similar to hMSCs as defined by the International Society for Cellular Therapy criteria [1]. Using an enzymatic method, we had a high recovery efficiency; in fact, we isolated an average of 4 × 10^5^ cells/cm^2^ by 4 cm^2^ arterial segments and, after 3 weeks of expansion, 250 × 10^6^ cells were achieved. This high output recovery may guarantee the possibility to isolate a cell amount needed for clinical application, limiting the necessity for a prolonged *in vitro* expansion that could alter stem cell features. In early passages (<3), the hC-MSCs showed intensive clonogenic ability, the 12 × 10^6^ freshly derived hC-MSCs adhered to plastic forming multiple colonies that rapidly became confluent, and the hC-MSCs were long-lived in culture and highly proliferative as demonstrated by their growth kinetics and immunofluorescence staining for ki-67.

In agreement with International Society for Cellular Therapy criteria, postmortem derived cells expressed the surface antigens commonly found in hMSCs – that is, CD44, CD73, CD90 and CD105 – and the lack of the expression of hematopoietic (CD14, CD34 and CD45) and vascular (vWF and CD31) lineages by flow cytometry confirmed the absence of blood and endothelial committed cells. Furthermore, triple flow cytometry immunostaining evidenced that more than 98.6% of CD34^–^/CD45^–^ cells expressed molecules commonly found in mesenchymal stromal/stem cells such as CD73 and CD105. Regarding the pericyte phenotype of hC-MSCs, 99.4% and 74% of CD44^+^/CD90^+^ coexpressed PDGF-rβ and CD146. In addition, they also expressed stemness molecules – that is, Stro-1, Oct-4 and Notch-1 – and HLA-G antigen, a well-known tolerogenic molecule [17] involved in the immunomodulatory activity of hMSCs. Immunofluorescence staining revealed a strong expression of Vimentin and Nestin; rare Neurofilament cells were positive. Nestin, a type VI intermediate filament, has been used to identify multipotent neural cells capable of differentiating along several neural lineages [30]. Because of the Nestin positivity and the presence of dendritic-like cells in inverted LM, we ruled out the possible contribution of a neural phenotype using additional neural markers such as NSE and S-100 that were completely negative. Apart from neural lineages, Nestin has been found expressed in normal arterial vasa vasorum as well as in endothelial cells of normal and pathological angiogenesis [31], and more recently in multipotent vascular stem cells of the rat [32]. Moreover, Nestin expression in hC-MSCs could be also related to the neural crest cell embryological origin of epiaortic segments and the aortic arch. Finally, the cells also expressed pericyte markers such as CD146, PDGF-rβ and NG2; this finding supports the evidence that pericytes may represent the hMSC *in situ* counterpart [33].

hC-MSCs retained the ability to express a set of genes associated with the embryonic stem cell marker and involved in the survival and proliferation/differentiation pathway including SOX2, c-KIT, the two isoforms of OCT-4 (380 bp, 308 bp) and KDR, while NOTCH-1 mRNA levels were lower. The high expression level of c-KIT and OCT-4 could be explained by hypothesizing that a subset of hC-MSCs had more ancestral characteristics.

However, the morphology and immunophenotype are not exclusive to give a cell population’s property of stemness: thus other features common to stem cells were investigated. As demonstrated previously [5], using ultralow attachment plates we selected from the hC-MSC cell population a stem cell subset that grows in suspension, forming embryoid body-like structures. Molecular analysis by RT-PCR showed expression of SOX2, OCT-4, c-KIT and KDR. One interesting characteristic related to the more primitive measure of progenitor cell activity is the ability of cells to reform colonies; accordingly, the clonogenic potential of single hC-MSCs was assessed at limiting dilution concentration and 8% of the total seeded wells displayed clonogenic properties. Nonclonogenic single cells had a ring-shaped morphology generated by the extrusion of long and thin cell processes that bent, forming circular profiles.

As other criteria for stem cells, it is generally considered necessary to demonstrate multipotency differentiating into adipocytes, osteocytes and chondrocytes. Adipogenesis resulted in a progressive increase in the size of multiple and confluent lipid-rich vacuoles, small dense mitochondria and intense endocytic activity into the cytoplasm and upregulation of PPARγ. Osteogenesis resulted in calcium deposition, electron-dense osteoid fibrillary matrix, needle-shaped hydroxyapatite crystals and increased expression of osteogenic differentiation genes (for example, Osteocalcin, Osteopontin and RUNX-2). Regarding chondrogenesis, cartilaginous differentiation was associated with alcianophilic, proteoglycan-rich extracellular matrix, glycogen accumulation and collagen type II mRNA expression and protein deposition in the cell cytoplasm.

Moreover, considering the vascular derivation of hC-MSCs, leiomyogenic and angiogenic abilities were also explored. The good propensity of hC-MSCs for leiomyogenic commitment resulted in the generation of myoid cells with peripherally arranged contractile filaments, subplasmalemmal linear densities and dense bodies. Similar to angiogenesis, VEGF-preconditioned hC-MSCs showed that these cells appeared connected by thicker projections forming an evident capillary-like network in a Matrigel assay. VEGF induction was accompanied by high expression of vWF and CD31, typical mature endothelium markers, supporting the commitment towards the endothelial cell lineage.

Apart from the multilineage differentiation ability [34], hMSCs are also capable of modulating immune responses, both *in vitro* and *in vivo*[35]. Immunomodulatory properties were initially reported using bone marrow-derived cells [36] and subsequently also using several alternative human sources [37-39]. In our study, to evaluate the immunomodulatory effects on immune system mononuclear cell proliferation, hMSCs were added to a mitogen-stimulated PBMC cell proliferation reaction. A previous study showed that hMSCs could silence T cells in the G_0_/G_1_ phase, which might be one of the possible mechanisms for the hMSC inhibitory effect on T cells [40]. We have assessed the hC-MSC immunosuppressive behavior by analyzing their ability to reduce proliferation of PHA-stimulated PBMCs. As reported by the PBMC cell cycle phase distribution, hC-MSCs exerted an inhibitory effect on activated PBMC proliferation, by reducing significantly PBMCs in the S and G_2_/M phases and blocking cells in the G_0_/G_1_ phase. Further investigation may confirm perspective applications in allogeneic conflicts.

## Conclusion

A cadaveric cell population with morphological, phenotypic and functional properties typical of mesenchymal stromal/stem cells survives in the vascular tissues after 4 days postmortem and following liquid nitrogen storage for more than 5 years. The isolated hC-MSCs are long lived in culture, highly proliferative and multipotent for their strong ability to differentiate in different mesengenic lineages; again these cells showed colony-forming ability, capability to form embryo-like bodies when grown in suspension and high immunosuppressive properties. Based on these results, in addition to easy accessibility, being noncontroversial, safety and abundant stem cell number, the procurement of hC-MSCs from cadaveric vascular tissues may be an alternative and inexhaustible reservoir of hMSCs for regenerative medicine and transplantation procedures.

## Abbreviations

bp: base pair; DMEM: Dulbecco’s modified Eagle’s medium; FBS: fetal bovine serum; FITC: fluorescein isothiocyanate; hC-MSCs: human cadaver mesenchymal stromal/stem cells; hMSCs: human mesenchymal stromal/stem cells; LM: light microscopy; mAb: monoclonal antibody; PBMC: peripheral blood mononuclear cell; PBS: phosphate-buffered saline; PCR: polymerase chain reaction; PDGF: platelet-derived growth factor; PE: phycoerythrin; PHA: phytohemagglutin; PPARγ: peroxisome proliferator-activated receptor gamma; RT: reverse transcriptase; Sm-GM2: smooth muscle growth medium-2; TEM: transmission electron microscopy; VEGF: vascular endothelial growth factor; vWF: von Willebrand factor.

## Competing interests

The authors declare that they have no competing interests.

## Authors’ contributions

SV and FA conceived and designed the experiments, performed the experiments, analyzed the data and wrote the paper. CC, FR and PLT performed the experiments and analyzed the data. MB and PP analyzed and interpreted data, and revised the paper. GP conceived and designed the experiments, analyzed the data, wrote the paper and revised the paper critically and gave final approval of the version to be published. All authors read and approved the final manuscript.
